# Tick-Borne Encephalitis with Hemorrhagic Syndrome, Novosibirsk Region, Russia, 1999

**DOI:** 10.3201/eid0906.030007

**Published:** 2003-06

**Authors:** Vladimir A. Ternovoi, Gennady P. Kurzhukov, Yuri V. Sokolov, Gennady Y. Ivanov, Vladimir A. Ivanisenko, Alexander V. Loktev, Robert W. Ryder, Sergey V. Netesov, Valery B. Loktev

**Affiliations:** * State Research Center of Virology and Biotechnology VECTOR, Koltsovo, Novosibirsk Region, Russi; † Novosibirsk State Medical Academy, Novosibirsk, Russia; ‡ First Municipal Clinical Infectious Hospital of Novosibirsk, Novosibirsk, Russia; § Stanford University, Palo Alto, California, USA; ¶ University of North Carolina, Chapel Hill, North Carolina, USA

**Keywords:** tick-borne encephalitis, TBE virus, hemorrhagic syndrome, flavivirus, emerging infection, Novosibirsk, dispatch

## Abstract

Eight fatal cases of tick-borne encephalitis with unusual hemorrhagic syndrome were identified in 1999 in the Novosibirsk Region, Russia. To study these strains, we sequenced cDNA fragments of protein E gene from six archival formalin-fixed brain samples. Phylogenetic analysis showed tick-borne encephalitis variants clustered with a Far Eastern subtype (homology 94.7%) but not with the Siberian subtype (82%).

Tick-borne encephalitis virus (TBEV) is one of many arthropod-borne viruses from genus *Flavivirus* (family: *Flaviviridae*) pathogenic to humans (*1*). Infection caused by TBEV is one of the most widespread natural foci infections in Russia; incidence varies from 5,593 to 10,298 cases annually and includes 89–166 deaths (*2*,*3*). The incidence of tick-borne encephalitis (TBE) increased sevenfold from 1974 to 1999 in Russia. The geographic distribution of the infection is uneven, with most illnesses occurring in the Siberian and Ural regions. In these regions, incidence is 10 to 30 times higher than in the Russian Far East region, where TBEV was discovered in 1937.

The major surface glycoprotein E is commonly used for studying phylogenetic relations of different TBEV strains. Analysis of the protein E sequence of 16 European and Asian TBEV strains showed clear segregation into three genetic subtypes, designated as European, Far Eastern, and Siberian (*4*). Genotyping of 75 TBEV strains typical for southern regions of Western Siberia showed that they differ considerably from European and Far Eastern strains (*5*–*7*). In reviewing published data, we found no substantial changes in the E gene in the Siberian subtype in 1981 to 1992 (*8*).

Our study was performed in the Novosibirsk Region of Western Siberia, where 243 to 534 cases of TBE are reported annually. We investigated retrospectively the first reported cases of lethal TBEV infection with hemorrhagic syndrome by using archival histologic samples. To determine the TBEV genotype that probably caused the hemorrhagic form of the infection, we sequenced cDNA fragments of protein E gene. We found that the TBEV strains that most likely caused the infection with hemorrhagic syndrome also carry unique mutations in protein E and belong to the Far Eastern genomic subtype.

## The Study

**Figure 1:**
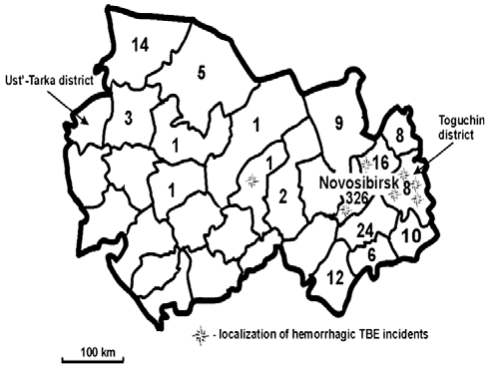
Distribution map of tick-borne encephalitis (TBE) cases by district, Novosibirsk Region, Russia, summer 1999. Case-patients were defined as persons who died from May 1 to August 15, 1999, and who had serologically confirmed (immunoglobulin M–positive test) tick-borne encephalitis infection.

In 1999, a total of 447 TBE cases confirmed by enzyme immunoassay were reported in Novosibirsk Region (Figure 1). Nine (2.0%) patients died; 72.9% of cases occurred in the city of Novosibirsk or its suburbs. The deaths of eight of these patients were associated with a pronounced hemorrhagic syndrome; symptoms included massive gastrointestinal bleedings and multiple hemorrhages in mucosa and internal organs. Four of the patients who died resided in the Toguchin District; the remainder lived in districts located near Novosibirsk (Figure 1). A total of 371 and 358 cases of TBEV infection confirmed by enzyme immunoassay and reverse transcriptase-polymerase chain reaction (RT-PCR) were reported in 2000 and 2001, respectively. In 2001, the mortality rate increased to 3.6%. Surveillance for TBEV during the summers of 2000 and 2001 identified no new cases of TBE with the hemorrhagic syndrome. This lack might be ascribed to the decrease in TBE incidence during this period, the change in the circulation of TBEV strains, and an unusual weather pattern during May and June.

We found no published reports describing a hemorrhagic disease caused by TBEV, although hemorrhagic manifestation is typical for tick-borne flaviviruses, including Omsk hemorrhagic fever virus (OHFV), Alkhurma virus, and Kyasanur Forest disease virus (*9*). Cases of OHFV occur occasionally in the Novosibirsk Region. Most cases result from the direct contact of a human with a muskrat, which was introduced in Siberia in 1928 (*10*,*11*), and most occur in the Ust’ Tarka District (Figure 1), located on the western border of the Novosibirsk Region (*12*). We found that hemorrhagic TBE emerged in the Toguchin district, located on the eastern border of the Novosibirsk Region, approximately 500 km from the Ust’ Tarka District. We found no cases of TBE in the central districts of the Novosibirsk Region, where the lakes and marshes make an unfavorable environment for the spread of this virus. The discovery of hemorrhagic TBE in the eastern part of the Novosibirsk Region is probably not related to the OHFV found in the far western part of the region.

By reviewing all available medical records, we retrospectively analyzed the eight cases of fatal hemorrhagic TBE infection. Diagnoses were confirmed serologically by testing for specific antiviral immunoglobulin M antibodies. All cases occurred in June and July 1999 after patients were bitten by ticks; the latent period was from 5 to 26 days (average 12.8). The ages of the patients ranged from 44 to 69 years. Disease onset included typical TBE clinical symptoms such as fever, myalgia, and malaise, followed by pronounced viral encephalitis accompanied by loss of consciousness, pareses, and paralyses. Hemorrhagic symptoms developed as massive gastrointestinal bleeding and local hemorrhages on mucosa and skin. The first sign of the hemorrhagic syndrome was erythrocytes in urine on day 7 of the infection, which is not usual for TBE infection. Common central nervous system manifestations occurred 3 days later. Patients died 2−3 days after the massive hemorrhagic syndrome developed, despite intensive treatment. The average time of death was 16 days after illness onset. Autopsies showed the pronounced hemorrhagic syndrome and viral encephalitis. Our retrospective screening of medical records from 1999 did not produce any evidence of a milder hemorrhagic syndrome in the rest of the case-patients.

Since hemorrhagic TBE has not been described previously, our objective was to determine the genotype of the virus. However, only six brain tissue samples from the fatal cases were available at the time this study was initiated. Archival samples of formalin-fixed brain tissue from the fatal hemorrhagic cases were collected in March 2000 and stored at −70°C. These samples had been stored for 8 to 9 months in 10% formaldehyde solution at room temperature at the pathology laboratory of the First Municipal Clinical Infectious Hospital of Novosibirsk. Viral RNA was isolated from formalin-fixed brain tissue by using the modified protocol described by Masuda et al. (*13*) and Coombs et al. (*14*). The RT-PCR system (GeneAmp RNA PCR Kit, Perkin-Elmer, Branchburg, NJ) was developed for detection of TBEV. RT-PCR primers were designed by using conserved DNA regions encoding gene E of TBEV, strain 205: 5′-TGCACACAYYTGGAAAACAGGGA-3′ (TBE913F), 5′-TGGCCACTTTTCAGGTGGTACTTGGTTCC-3′ (TBE1738R). The sense primer 5′-CAGAGTGATCGAGGCTGGGGYAA-3′ (TBE1192F) and antisense primer 5′-AACACTCCAGTCTGGTCTCCRAGGTTGTA-3′ (1669R) were used for second round of PCR.

Nucleotide sequences of E gene fragment PCR products (1,192–1,661 bp) of TBEV strains were determined by using a Beckman sequencing kit and Beckman CEQ2000XL DNA Analysis System (Beckman Coulter, Inc., Fullerton, CA) according to manufacturer’s instructions. These samples were used to isolate RNA and generate cDNA fragments corresponding to positions 1,192−1,669 bp of protein E gene by using nested RT-PCR. The amplification products were sequenced. We submitted these sequences to GenBank (accession nos. AF540029–AF540034).

**Figure 2:**
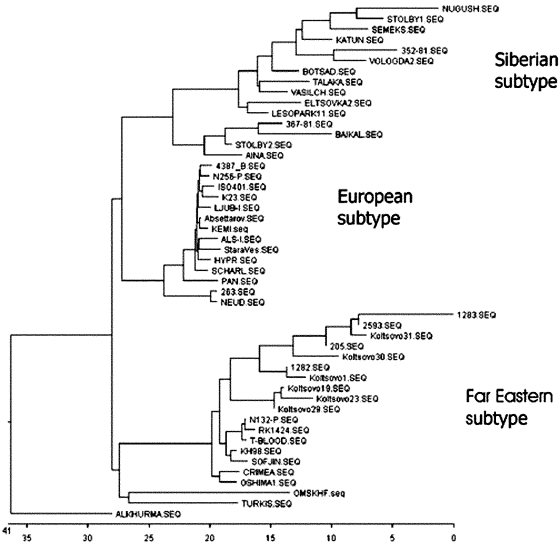
Phylogenetic tree illustrating the genetic relationship of hemorrhagic tick-borne encephalitis virus variants with prototype strains from other subtypes of the virus (generated for nucleotide sequences of protein E gene, fragment 1,192−1,669). Nucleotide and deduced amino acid sequences were aligned by using Clustal X and MEGALIGN v4.04. Phylogenetic tree was constructed by MEGA v2.1. GenBank data were used for comparison of strains of tick-borne encephalitis viruses with another tick-borne flaviviruses. The list of the sequences used (locus name and accession no.) is: 263, 4387/B7, 973, Absettarov, Als.I, Hypr, Iso 40, K23, Kem I, Ljub.I, N256, Neudoerfl, Pan, Scharl, Stara Ves, Turkish, Western subtype Mandl (U27491, X76608, AF241774, AF091005, AF091007, U39292, AF091009–12, AF091014, U27495, AF091015, AF091017, AF091018, L01265, NC001672); Crimea, Hehcir, KH98-10, N132, O-I-1, Oshima C-1, Oshima-5-10, RK1424, Sofiin, T-blood, Latvia (AF091008, AF229363, AB022297, AF091013, AB022292, AB022294, AB001026, AF091016, X07755, AF091019, AJ010192); 352-81, 367-81, Aina, Baikal-4, s Botsad-1, Katun-3, Nugush-2, Semeks, Stolby-1, Stolby-4, Talakan-4, Vasilchenko, Vologda-2 (AF224667, AF241773, AF091006, AF229362, AF224662, AF236055, AF224663, AF224665, AF224666, AF231807, AF241772, AF069066, AF229364); Omsk hemorrhagic fever virus (Omskhf) (X66694); and Alkhurma virus (NC004355).

The homologic values between the nucleotide sequences of protein E fragment of hemorrhagic TBEV, Siberian, and Far Eastern TBEV subtypes are shown in Table 1. The nucleotide sequences of hemorrhagic TBEV strains show approximately the same degree of homology (82%) with protein E gene of different strains of Siberian subtype (strains Lesopark-11, Eltsovka-2, and Vasilchnenko isolated near Novosibirsk) and 94% homology with the Far Eastern subtype. The typical phylogenetic tree with support values is shown in Figure 2. Because hemorrhagic TBEV strains clustered with the Far Eastern subtype of TBEV, we associated them with the Far Eastern subtype.

**Table 1:** Homology between the nucleotide acid sequences of prototype strains for three subtypes of tick-borne encephalitis virus, Omsk hemorrhagic fever virus, and hemorrhagic tick-borne encephalitis virus^a^

Viruses	European subtype (subtype 1) (%)		Far Eastern subtype (subtype 2) (%)			Siberian subtype (subtype 3) (%)			Omsk hemorrhagic fever virus
	Neud	KemI	Sofiin	Oshima1	Crimea	Botsad	Eltsovka	Lesopark	Omskhf
Neud	100.00	98.06	81.94	82.78	82.50	84.72	83.89	84.72	80.28
KemI	98.06	100.00	81.39	82.50	81.94	83.89	83.06	83.89	80.00
Sofiin	81.94	81.39	100.00	95.83	94.44	85.28	84.72	85.28	77.78
Oshima1	82.78	82.50	95.83	100.00	97.50	85.83	85.28	85.83	77.22
Crimea	82.50	83.06	94.44	97.50	100.00	86.67	85.56	86.67	78.33
**Koltsovo 1**	**81.67**	**81.11**	**99.72**	**96.11**	**94.72**	**85.00**	**84.44**	**85.00**	**78.06**
**Koltsovo 19**	**80.28**	**79.44**	**92.50**	**93.61**	**93.06**	**83.33**	**82.22**	**83.33**	**76.67**
**Koltsovo 23**	**78.61**	**78.06**	**91.39**	**92.22**	**91.94**	**82.50**	**81.39**	**82.50**	**75.00**
**Koltsovo 29**	**80.83**	**80.00**	**92.78**	**93.89**	**93.33**	**83.61**	**82.50**	**83.61**	**77.50**
Botsad	84.72	83.89	85.28	85.83	86.67	100.00	97.78	98.89	78.89
Eltsovka	83.89	81.94	84.72	85.28	85.56	97.78	100.00	97.78	78.06
Lesopark	84.72	83.89	85.28	85.83	86.67	98.89	97.78	100.00	78.06
Omskhf	80.28	80.00	77.78	77.22	78.33	78.89	78.06	78.06	100.00

^a^Viruses shown in boldface are the hemorrhagic variants of tick-borne encephalitis virus.

The amino acid sequence of hemorrhagic TBE has higher homology with the Far Eastern subtype (98%) than with the Siberian subtype (96%). We identified 13 different amino acid mutations in six sequenced fragments of protein E (Table 2). These mutations were not previously described for TBEV; however, most of them (except 121C, 244L, and 249A) were found among other flaviviruses. Cysteine 121 is highly conserved among all flaviviruses since it is involved in maintaining the protein E 3D structure through a disulfide bridge with cysteine 92. Substituting cysteine 121 for glycine in the Koltsovo 31 TBEV variant might cause dramatic changes in protein E structure and function.

**Table 2:** Mutations in the amino acid sequence of protein E of hemorrhagic variants of TBEV^a^

Amino acid, position	TBEV, 205	Mutation in hemorrhagic TBEV	Variants of hemorrhagic TBEV	Mutations in other flaviviruses^b^
121	Cys	Gly	Koltsovo 31	
128	Thr	Ala	Koltsovo 19	Val,^c^ Ser,^d^ Ala,^e^ Ile^f^
129	Gly	Arg	Koltsovo 31	Leu^d^
138	Val	Ala	Koltsovo 19	Thr,^c,g^ Gln,^d^ Lys,^e,f,h–k^ Glu^l,m^
141	Val	Asp	Koltsovo 31	Val,^c,d,g^ Glu,^e,f, h,i^ Ser,^j^ Thr^k,l,m^
170	Glu	Gly	Koltsovo 29	Gly,^d^ Pro,^e,f,h–j,m^ Ser,^k,l^
171	Arg	Lys	Koltsovo 1	Lys,^c,g^ Ser,^d,f,h,i,m^ Ala,^e^ Thr,^j,k^ Ile^l^
189	Ala	Ser	Koltsovo 23	Thr,^g^ Gln,^d^ Arg,^e,f,h–1^ Gly^m^
223	Leu	Trp	Koltsovo 23	
224	Ala	His	Koltsovo 23	Ser,^g^ Thr,^d^ Leu,^e^ Asn,^h,i^ Pro^j–m^
244	Phe	Cys	Koltsovo 30	
245	Gly	Val	Koltsovo 29	Glu,^d–f,h,i^ Lys^j–m^
249	Ala	Arg	Koltsovo 30	

^a^TBEV, tick-borne encephalitis virus. ^b^Alignment of protein E sequences was carried out with data from the Protein Families database (available from: URL: http://www.sanger.ac.uk/Software/Pfam/). ^c^Powassan virus, strain LB (Q04538). ^d^Yellow fever virus, strain 17D (P03314). ^e^Murray valley encephalitis virus (P05769). ^f^Japanese encephalitis virus, strain CH2195 (O09754). ^g^Kyasanur forest disease virus (SWISS-PROT Q82951). ^h^Kunjin virus, strain MRM61C (P14335). ^i^St. Louis encephalitis virus, strain MS1-7 (Q88788). ^j^Dengue virus type 1 (O10246). ^k^Dengue virus type 3 (P27915). ^l^Dengue virus type 2, strain 16681 (O09234). ^m^Dengue virus type 4 (Q88668).

## Conclusions

Long-term surveillance in Siberia indicates that persons with TBEV infection have relatively mild fever; death occurs in 1% of patients (*2*). The high incidence of TBE in Western Siberia is attributed to the active circulation of Siberian TBEV variants (*3*,*6*,*7*). By sequencing a fragment of protein E, we found these variants grouped to a separate subtype (Siberian). Overall, 75 protein E sequences of TBEV variants isolated in 1952 to 2002 in different regions of Russia were published. Of these virus isolates, 46 were collected directly in the suburbs of Novosibirsk (*8*). Our study showed that Siberian variants have approximately 78% to 81% homology in protein E genes with the Far Eastern TBEV subtype.

Our sequencing data showed that the genome of hemorrhagic TBEV variants differs from already known Siberian TBEV and OHFV strains. Variants of hemorrhagic TBEV show the highest degree of homology with the Far Eastern subtype, represented by prototype strains 205 and Sofiin. This finding supports our hypothesis that a relationship exists between the occurrence of unusual clinical disease and emergence of new TBEV variants in the Novosibirsk Region.
